# P3b Reflects Periodicity in Linguistic Sequences

**DOI:** 10.1371/journal.pone.0051419

**Published:** 2012-12-10

**Authors:** Sascha Otterbein, Cornelius Abel, Linda V. Heinemann, Jochen Kaiser, Maren Schmidt-Kassow

**Affiliations:** Institute of Medical Psychology, Goethe-University, Frankfurt, Germany; University Medical Center Groningen UMCG, The Netherlands

## Abstract

Temporal predictability is thought to affect stimulus processing by facilitating the allocation of attentional resources. Recent studies have shown that periodicity of a tonal sequence results in a decreased peak latency and a larger amplitude of the P3b compared with temporally random, i.e., aperiodic sequences. We investigated whether this applies also to sequences of linguistic stimuli (syllables), although speech is usually aperiodic. We compared aperiodic syllable sequences with two temporally regular conditions. In one condition, the interval between syllable onset was fixed, whereas in a second condition the interval between the syllables’ perceptual center (p-center) was kept constant. Event-related potentials were assessed in 30 adults who were instructed to detect irregularities in the stimulus sequences. We found larger P3b amplitudes for both temporally predictable conditions as compared to the aperiodic condition and a shorter P3b latency in the p-center condition than in both other conditions. These findings demonstrate that even in acoustically more complex sequences such as syllable streams, temporal predictability facilitates the processing of deviant stimuli. Furthermore, we provide first electrophysiological evidence for the relevance of the p-center concept in linguistic stimulus processing.

## Introduction

Stimulus periodicity of acoustic stimuli is assumed to facilitate attention allocation to predictable points in time. According to the Dynamic Attending Theory (DAT) [1] internal oscillations may synchronize with external oscillators over time. In contrast to randomly emerging stimuli, any periodic signal of a given frequency and phase can readily be anticipated by entrainment of internal oscillations, resulting in stimulus-driven attending [2]. Synchronization directs attention to those points in time when the prospective manifestations of a relevant signal are expected to occur. At the neuronal level, Lakatos et al. [3] have found enhanced attention to be associated with electrophysiologic oscillations entrained to relevant stimuli patterns. Stimulus-induced expectancies should thus facilitate the efficient allocation of cognitive resources.

In humans, event-related potentials (ERPs) can be used to test attentional processing during stimulus perception and evaluation. The P3b subcomponent is a positive ERP deflection at central and parietal electrode sites that usually peaks 300–500 ms after stimulus onset. The attention-related component has been probed recurrently in both the visual and the auditory modality by employing variants of the traditional two-stimulus oddball paradigm in which infrequent targets are interspersed in a sequence of standard stimuli. The P3b has been related to context updating, i.e. changing the mental model of the environment in order to generate an appropriate response, and subsequent memory storage (see [4]), for a review). P3b amplitude and peak latency are hypothesized to reflect the amount of attentional resources engaged and should thus vary with stimulus temporal predictability. Although other factors have been proposed to influence the P3b such as stimulus probability, we focused on temporal predictability as this was the critical manipulation in the current study while all other factors were kept constant. The effect of temporal predictability on attention allocation has been assessed in electrophysiological studies employing tone sequences. For instance, Schmidt-Kassow et al. [5] have shown that attentional processing is affected by temporal predictability. Utilizing tone sequences in a P3b paradigm they found a larger P3b amplitude and shorter peak latency for isochronously presented compared to chunked or randomly presented stimuli. Task performance, i.e. detection of deviant tones was also increased for the regular pattern sequences. Similarly, other studies have found an enhanced P3 in regular timing contexts (e.g., [6]–[8]). Recently Schwartze et al. [9] have dissociated the mechanisms of pre-attentive and attention-dependent temporal processing. Temporal regularity did not modulate mismatch negativity, P3a or reorienting negativity in a non-attending context. However, if participants directed attention towards the tone stimuli, deviant tones that were embedded in a temporally regular stream elicited an enhanced P3b. In conjunction with an unaffected earlier N2b this suggests that a regular temporal structure does not contribute to the detection of a deviant, but facilitates later memory processing and model updating [4].

The present experiment assessed whether temporal cues also facilitate the processing of auditory streams with more complex, linguistic stimuli. The influence of temporal predictability on linguistic processing is not well understood. During auditory language processing the auditory input stream needs to be segmented appropriately. In this context, the perception of rhythm in speech utterances is crucial, in particular in first language acquisition [10], [11]. Rhythm in speech refers to the systematic organization of a sequence of events in time. Any perceived rhythmic pattern in speech is defined by suprasegmental cues as, e.g., the relative prominence of a syllable (i.e., stress). Syllable sequences of alternating prominence constitute rhythmic patterns that are potentially perceived as regular. However, the importance of periodicity in contrast to perceptual regularity in linguistic processing is less clear. Although previous research has revealed some rhythmical constraints on stress timing in speech production (e.g., [12]), only few acoustic correlates of periodicity have been found in the physical speech signal to date. This suggests that temporal regularity in speech is a mere perceptual phenomenon. Hence, during linguistic processing, the language system should rather focus on extracting regularities in irregularly timed sequences than analyzing periodic events as such events should practically not exist.

The current study investigated whether physical regularity, i.e. periodicity, in linguistic sequences leads to a processing benefit comparable to previous findings for tonal sequences. Segmenting linguistic sequences into regular timing patterns seems more complex than aligning simple tones. To enable synchronization to a stream of syllables it might suffice to align the syllables according to their physical onset, which means to arrange the stimuli using a constant stimulus onset asynchrony (SOA). A more elaborated approach would first identify the beat in a language signal and then utilize this putatively optimal time point to align the stimuli. Here, we used the perceptual center (p-center) for this purpose. The p-center of any acoustic event is defined as the perceived moment of its occurrence, which is commonly non-congruent with the physical signal onset [13], [14]. In linguistic stimuli, most commonly, the syllable nucleus onset, i.e., the onset of the vowel of the stressed syllable is considered as the p-center [15].

The P3b component as a sensitive marker for the amount of attentional resources should vary as a function of temporal predictability. We expected larger amplitudes and shorter latencies of the P3b component for the isochronous timing conditions than for the irregular timing condition. We expected the effects to be localized at central and parietal electrode sites [5]. Considering behavioral performance, accuracy was expected to be highest for the isochronous timing conditions. Moreover, we hypothesized that the p-center is the most profound parameter for aligning speech. We therefore expected differences between the two isochronous conditions with more efficient allocation of attentional resources and correspondingly higher P3b amplitude and shorter latency for the p-center aligned sequences than for the sequences aligned to physical stimulus onset.

## Materials and Methods

### Subjects

Thirty right-handed volunteers (13 males) with unimpaired hearing participated in this study. Four datasets were discarded (see below). The age of the remaining subjects ranged from 20 to 26 years (M = 22.6 y, SD = 1.7 y) and was matched between sexes. All subjects had normal or corrected-to-normal vision and no history of psychiatric or neurological disorder.

### Ethics Statement

The study was approved by the Ethics Committee of the University of Frankfurt Medical Faculty. All subjects were aware of the aims of the study and gave informed written consent.

### Stimuli

Syllables of variable durations were generated with MBROLA [16]. Its high-quality diphone-based speech synthesis algorithm allowed for rigorously controlling phonemes and prosody. The rationale for synthesizing syllables was to vary the p-center without altering other parameters such as phoneme duration, intensity, or pitch. Adopting the introduced p-center model from Janker [15], the p-centers of the generated syllables were manually set to the respective vowel onset by the senior author (MSK) using the Praat software package for analysis of speech in phonetics [17].

The initial bilabial plosive/p/and a subsequent vowel/a/or/ae/were common to all synthesized syllables. We varied the onset of the vowel (i.e., p-center) by inserting none, one, or two phonemes between the initial plosive and the vowel; in particular, a lateral approximant/l/, either alone or in combination with a preceding fricative/f/. Taking two vowel qualities into consideration, a total of six distinguishable syllables were synthesized (/pa:/,/pla:/,/pfla:/, and/pε:/,/plε:/,/pflε:/), see Table 1 for syllable durations and p-center. Audio volume levels were normalized.

**Table 1:** Stimuli.

Syllable	Duration [ms]	p-center [ms]	Syllable	Duration [ms]	p-center [ms]
pa:	228.3	81	pε:	226.8	86
pla:	278.2	136	plε:	278.5	138
pfla:	328.4	188	Pflε:	328.8	189

**Figure 1:**
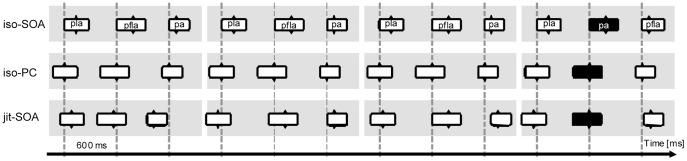
Stimulus alignment for the three timing conditions. Critical targets are marked in black. *iso-SOA* = isochronous physical syllable onsets; *iso-PC* = isochronous vowel onsets; *jit-SOA = *jittered physical syllable onsets.

Three syllables with an identical vowel but with temporally varying p-centers were fully permutated thereby arranging them into six distinct syllable triplets (e.g., pa - pla - pfla). Each possible triplet served as a template for assembling a total of 216 trials of twelve syllables each. Upon compilation, all trials comprised four identical (i.e., standard) syllable triplets rendering them structurally consistent. Half of the trials were then altered to deviate by swapping the last two syllables of the sequence. Hence, the eleventh syllable could constitute a deviant (see Figure 1; see supporting information for sound examples).

To examine the effects of temporal context, the syllable sequence trials were grouped into three timing conditions, namely isochronous physical syllable onsets (*iso-SOA*), isochronous vowel onsets, i.e., p-centers (*iso-PC*), and jittered physical syllable onsets (*jit-SOA*) (Figure 1). In the *iso-SOA* timing, syllables were aligned to their physical onsets with a constant SOA of 600 ms. In the *iso-PC* timing, the onset of the vowel established the basis for stimulus alignment with a 600 ms interval between two successive p-centers. The 600-ms interval is close to the preferred spacing of events, i.e., a pace that is experienced as natural and that can be easily synchronized with [18]. In addition, a stimulus rate of 600 ms corresponds to the inter-stress intervals found across the languages investigated by Dauer [19]. In the isochronous conditions attention should entrain with the 600 ms time points, i.e. a maximum of focal attention should coincide either with the physical onset of each syllable or its p-center. In the *jit-SOA* timing the inter-stimulus intervals among all syllables were pseudo-randomized: a jitter randomly chosen out of a vector of 11 linearly spaced numbers from −175 to +175 (SD: 116.08 ms) was added to an SOA of 600 ms. Here, it should not be possible to predict the next stimulus occurrence. Stimuli were presented via headphones (AKG K271, HARMAN International Industries, Stanford, USA), using the software package Presentation v14.8, (Neurobehavioral Systems, Albany, USA).

### Procedure

Prior to the actual experiment, participants were accustomed to the syllable sequences and the task in a short training session of five trials. The volume of the auditory stimulation was ensured to be properly adjusted to a comfortable listening level so that the subjects could discriminate the stimuli. An asterisk at the center of the screen marked the beginning of each trial, alerting the participants to the forthcoming stimuli. The asterisk remained on the screen throughout presentation of the sequence. Subjects were instructed to fixate the asterisk without blinking. The auditory stimulation began 500 ms after presentation of the asterisk.

Participants decided for each syllable sequence if the pattern was consistent throughout the trial or if the syllable succession changed. They were not explicitly instructed to respond as fast as possible. Instead, participants were asked to enter their response (consistent/standard or inconsistent/deviant) by pressing the corresponding key after being visually prompted with a question mark for 1000 ms. The key assignment to either the left or the right index finger was counterbalanced across subjects. Each trial ended with a blank screen with a variable duration of 1000 to1500 ms that allowed the participants to blink and to prepare for the next syllable sequence.

A total of 216 (3 timing conditions (iso-PC, iso-SOA, jit-SOA)×2 conditions (standard vs. deviant)×6 triplet permutations×2 vowel qualities×3 repetitions) trials were shuffled for each participant. Constrained randomization ensured that there were no more than two trials of the same timing twice in direct succession and no more than three consecutive standards or deviant trials. The trials were binaurally presented in 12 balanced blocks of 18 syllable sequences. Each 3.5 min block ended with an easy visual task that was intended to enhance alertness and to keep alpha band power to a minimum throughout the experiment. Data from this task was not analyzed. Subjects counted up cartoon characters in a series of four randomly assorted still images showing 0–10 sheep for 4000 ms each. A prompt displayed the correct total or a sum that marginally differed in either direction for 3000 ms. In case the count matched the displayed number, the participant was asked to press any key, else none. A facultative short rest was offered after each block. The participants did not receive feedback on their performance throughout the session, which took about two hours including electrode attachment, training, and breaks.

### Electrophysiological Recording and Analysis

The recordings took place in an electrically shielded and sound-attenuated chamber (Industrial Acoustic Company GmbH, Niederkrüchten, Germany). The stimulation monitor used to present a fixation asterisk, the prompt, and the still images was placed outside the cabin behind an electrically shielded window.

**Figure 2:**
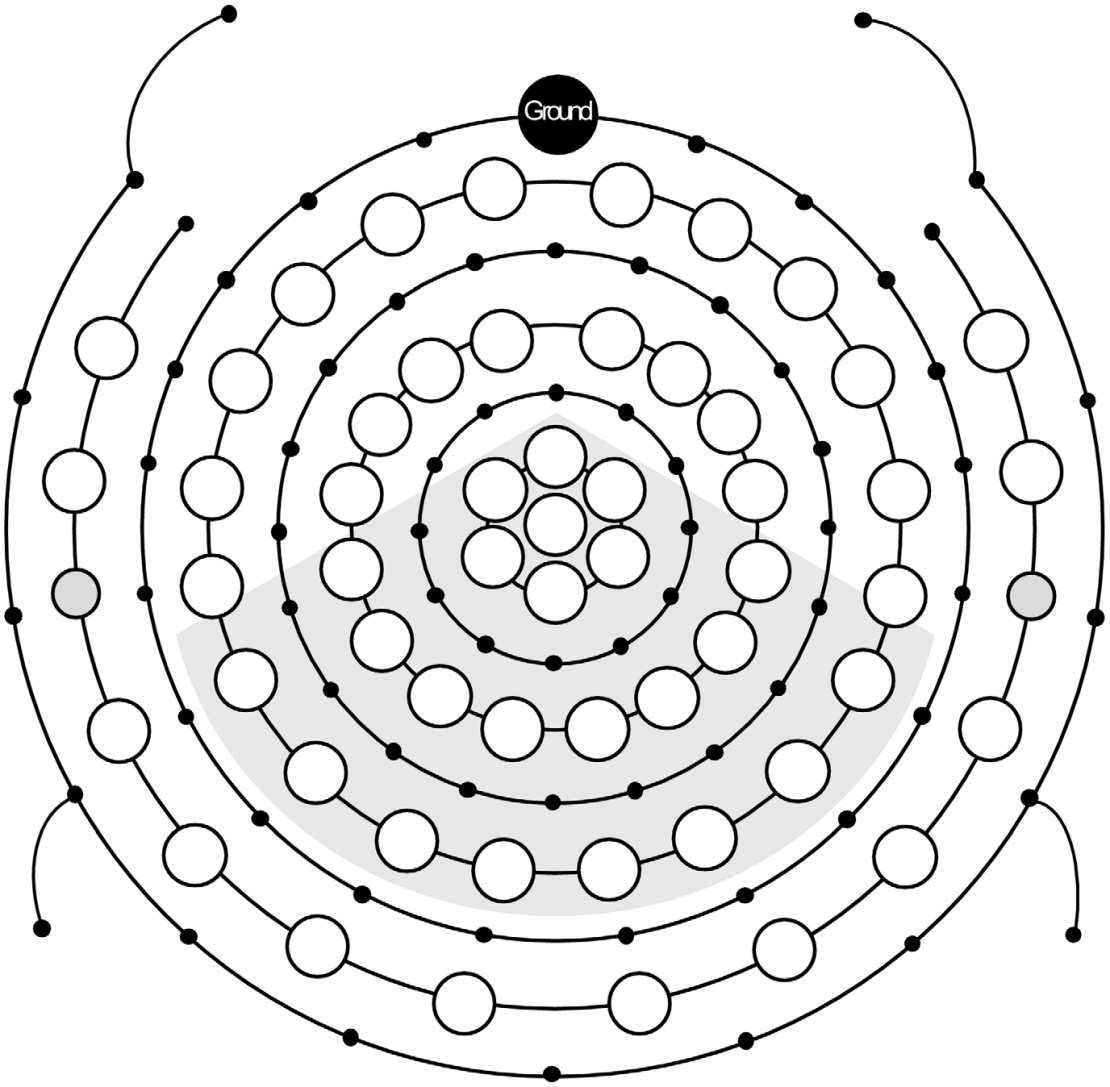
Electrode setup. 23 electrodes of interest are shaded in grey.

EEG was recorded using a QuickAmp amplifier (Brain Products, Munich, Germany) and Braincap electrode caps (Falk Minow Services, Munich, Germany) with 65 electrodes mounted in an elastic cap in concentric shapes uniformly covering the whole head. A subset of eight electrodes was mounted infra- and supraorbitally to monitor eye blinks and movements. All channels were recorded with an average reference, a forehead ground and impedances of less than 7 kΩ. The EEG was digitized with a sampling rate of 500 Hz and an anti-aliasing filter of 135 Hz. Based on previous work [5], 23 out of the 65 electrodes constituted the central and parietal region of interest as depicted in Figure 2. This ROI underwent statistical analysis.

The preprocessing of the EEG data and the statistical analyses were carried out in MATLAB (The Mathworks, Natrick, USA) making use of the open source FieldTrip toolbox for EEG/MEG analysis [20] developed at the Donders Institute for Brain, Cognition, and Behavior (Nijmegen, The Netherlands). Upon inspection one excessively noisy data set was removed. Artifacts of the ROI electrodes were detected by means of thresholding the z-transformed value of the preprocessed raw data. A band-pass filter (110–140 Hz) was applied, Hilbert analytic amplitude extracted and the z-scores calculated. The z-score cutoff was set to 6 across data sets. To identify eye artifacts the data underwent independent component analysis (ICA) decomposition. All components were correlated with signals at the orbital channels. Components with correlations higher than the mean correlation plus 3 standard deviations were subtracted from the ROI electrode data.

EEG data were segmented into 1200 ms epochs (200 ms pre- through 1000 ms post-stimulus) spanning the critical eleventh syllable of a trial. All epochs were time-locked to the physical onset of each syllable irrespective of its timing condition. For each epoch, a 100-ms pre-stimulus baseline correction was performed. To reject non-alert participants, mean power estimates across the full epoch were calculated for the alpha frequency band between 8 and 14 Hz based on a single Hanning taper (M = 3.26, SD = 2.68). Three subjects with an outlying alpha-band power >8.62 (µV^2^/Hz) were removed from further analysis.

ERPs were then computed separately for standards and deviants in each timing condition by averaging the waveforms across 23 central and parietal ROI electrodes (see Figure 2).

Only trials with correct responses were considered. Thus, for the 26 remaining participants, on average 77.8% (SD = 10.3%) of the 36 epochs per condition were retained after artifact rejection. Difference waves were calculated by subtracting the ERPs to standard stimuli from those to the deviants. Prior to the analysis, the ERP and difference wave data were filtered with a band pass of 0.4 Hz - 8 Hz and re-referenced to average mastoids.

Based on visual inspection of the data and on previous experiments [5], a time window from 450 to 800 ms was used to analyze the P3b. For statistical analysis, the P3b mean amplitudes (means were calculated across this time window) were subjected to a repeated-measures ANOVA at central and parietal electrodes with the two within-subject factors timing (*iso-PC*, *iso-SOA*, *jit-SOA*) and deviance (*standard*, *deviant*). Bonferroni follow-up tests were conducted where appropriate. When evaluating effects with more than one degree of freedom sphericity was tested with Mauchley’s test and corrected where appropriate [21]. To uncover potential peak latency differences, a peak amplitude analysis of the P3b component was conducted employing the within-factor *timing*. For each subject the maximum positive deflection relative to the pre-stimulus baseline was determined for the 450–800 ms time interval.

## Results

### Behavioral Data

**Figure 3:**
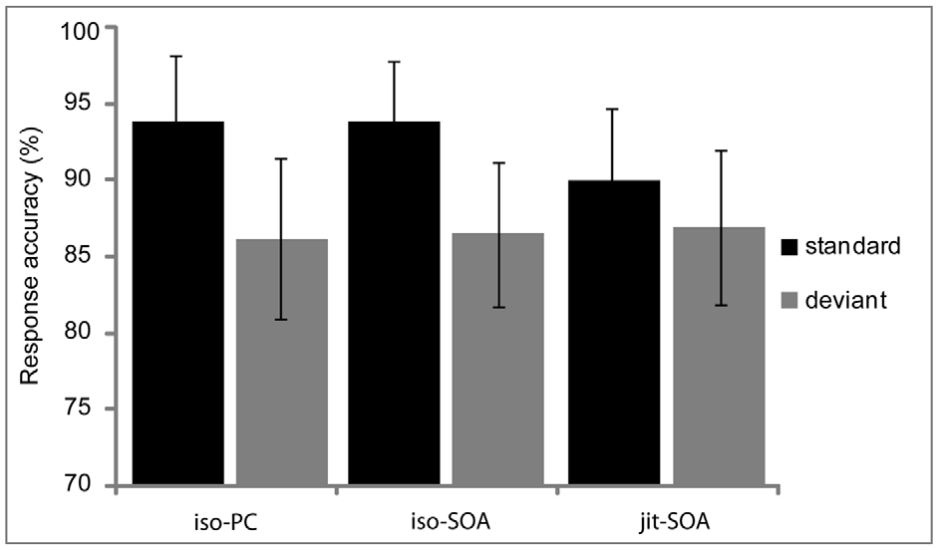
Response accuracy. Error bars indicate the SD.

The overall responses of the participants were accurate (M = 89.6%, SD = 9.6%), indicating that they paid attention to the syllable sequences. Testing response accuracy (see Figure 3, Table 2) with a two-way repeated-measures ANOVA yielded a main effect of *timing* (MS = 53, F[2,50] = 3.91, p<0.05), a main effect of *deviance* (MS = 1429, F[1], [25] = 8.39, p<0.01), and an interaction of *timing×deviance* (MS = 86, F[2,50] = 4.27, p = 0.019).

**Table 2:** Response accuracy.

Response accuracy [%]	iso-PC		iso-SOA			jit-SOA		
	Mean	SD		Mean	SD		Mean	SD
standard	93.9	7.9		93.9	7.2		90.0	8.3
deviant	86.2	10.4		86.5	9.4		86.9	10.1

Post-hoc testing the interaction effect by applying the Bonferroni test revealed that performance was better for standards than deviants trials in both *isochronous* timing conditions (*iso-SOA*: t[25] = 3.56, p<0.001; *iso-PC*: t[25] = 3.49, p<0.001). There was no accuracy difference in the *jit-SOA* condition (p = 0.24). Furthermore, post-hoc tests revealed significant differences for standard trials between iso-PC and jit-SOA timing (t[25] = 3.07, p<0.001) as well as between iso-SOA and jit-SOA timing (t[25] = 3.53, p<0.001) but not between isochronous timing conditions (p = 0.9). There were no accuracy differences between timing conditions for deviant trials (p>0.3).

### Event-related Potentials

#### P3b

**Figure 4:**
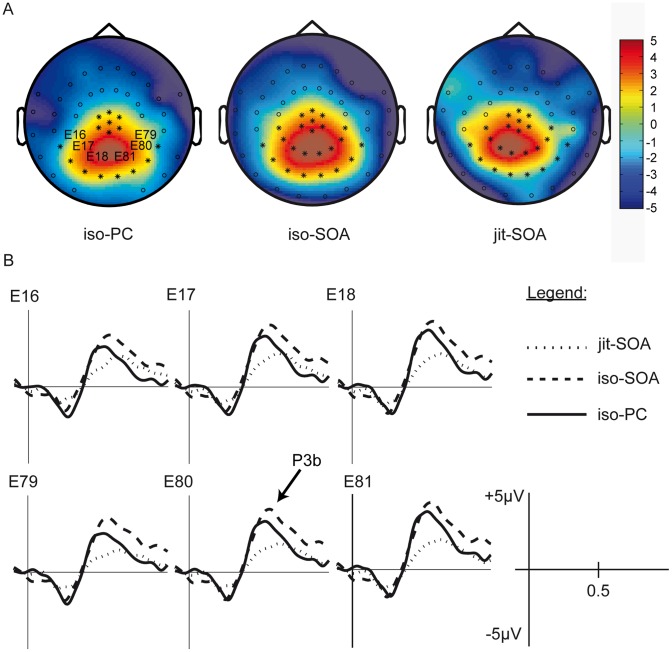
P3b response. Panel A: P3b topography for each timing condition. ROI electrodes are marked with asterisks. Panel B: P3b difference waves at 6 representative ROI electrodes for each condition.

**Figure 5:**
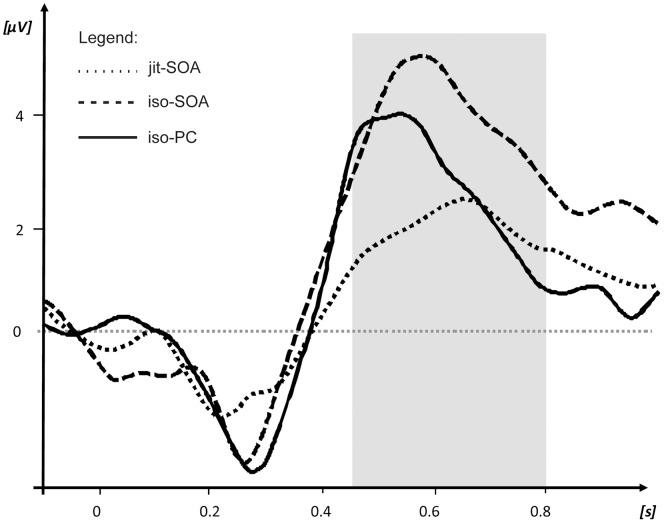
P3b difference waves averaged across 23 ROI electrode sites in the time window of 450–800 ms.

The ANOVA revealed a main effect of *deviance* (MS = 337.61, F[1], [25] = 51.41, p<0.001), with higher amplitudes for deviant trials (M = 3.95 µV, SD = 3.07 µV) than for standard trials (M = 1.01 µV, SD = 2.2 µV). In addition, there was a main effect of *timing* (MS = 56.36, F[2,50] = 17.32, p<0.001) and an interaction *timing×deviance* (MS = 15.52, F[2,50] = 3.02, p = 0.05), see Figure 4 and Figure 5.

Post-hoc comparisons for the interaction *timing x deviance* revealed differences for the following pair-wise contrasts: *iso-PC* standard vs. *iso-PC* deviant (t[25] = −3.69, p = 0.001), *iso-SOA* standard vs. *iso-SOA* deviant (t[25] = −6.28, p<0.001), *jit-SOA* standard vs. *jit-SOA* deviant (t[25] = −3.54; p = 0.001), deviant *iso-PC* vs. deviant *jit-SOA* (t[25] = 3.74, p<0.001), deviant *iso-SOA* vs. deviant *jit-SOA* (t[25] = 5.57, p<0.001), Table 3. In contrast, there were neither amplitude differences between the standard trials of all three timing levels nor between the deviant trials of the *iso-PC* and *iso-SOA* timing conditions. Bonferroni-corrected alpha-level was 0.0056.

**Table 3:** Mean amplitudes for each timing condition in the time window 450–800 ms.

Mean amplitude [µV]	iso-PC		iso-SOA		jit-SOA	
	Mean	SD	Mean	SD	Mean	SD
standard	0.94	2.06	0.53	2.02	0.23	2.07
deviant	4.66	3.33	5.22	2.79	2.15	2.68

The repeated-measures ANOVA for latency yielded a main effect of *timing* (F[2,50] = 8.8, p<0.001). The post-hoc comparison showed that the P3b elicited by *iso-PC* timing deviants peaked earlier (M = 524.7 ms, SD = 62.9 ms) than the P3b elicited by the *iso-SOA* (M = 594.2 ms, SD = 65.9 ms, t[25] = −4.43, p<0.001 ) or the *jit-SOA* timing condition (M = 591.2 ms, SD = 88.0 ms, t[25] = −3.7, p = 0.001, ). There was no significant peak latency difference between the *iso*-*SOA* and the *jit-SOA* timing condition. Bonferroni-corrected alpha-level was 0.016.

## Discussion

This study used auditorily presented syllables to investigate the modulation of attentional processing of basic language stimuli by periodicity. Stimuli were presented in sequences of four syllable triplets that could either be identical or deviate at the last but one syllable of the sequence. Temporal predictability was manipulated by contrasting an irregular timing condition with two isochronous timing conditions aligned either with physical stimulus onset or with the p-center.

As expected, performance differed between timing conditions. Standard sequences were detected more accurately in both isochronous timing conditions than in the irregular condition. In contrast, detection of deviants did not depend on stimulus predictability. Looking at ERPs, deviants embedded in the stimulus stream reliably elicited P3b components in all timing conditions. However, compared with deviants of irregularly aligned syllable streams, temporally predictable stimuli gave rise to larger P3b amplitudes. This indicates enhanced evaluation of temporally predictable compared with irregular stimulus streams.

These findings indicate that attention is sensitive to task-irrelevant timing properties (cf. [9], [22], as the observed influence of temporal regularity was independent of explicit attention to time. The observed modulation of the P3b component extends previous findings showing more efficient processing of tonal stimuli occurring at expected time points (e.g., [23], [8], [5], [9]. These studies have interpreted the P3b as an index for the quality of stimulus-driven synchronization, i.e., periodic stimulus presentation supports the processing of formal stimulus characteristics. Although the present data cannot prove that participants synchronized to the syllable stream which could have been demonstrated by requiring a finger tapping response, the observed effects support the account of a dynamic allocation of attention as postulated by the DAT [1]. According to this model attention should be shifted to prospective points in time when relevant events are expected to occur. This should lead to an increased allocation of cognitive resources for stimulus processing resulting in the facilitated detection of a target. We found that attending to syllable triplets that were consistent across an entire sequence was less demanding in the regular timing conditions. In contrast, when synchronization was not possible, syllable occurrence could not be temporally predicted and processing demands were higher. Here, we showed that this phenomenon is not restricted to a single difference in stimulus characteristics such as sound frequency, but is also effective for more complex stimuli such as linguistic sequences.

One might wonder about the discrepancy between behavioral and ERP results. We argue that behavioral and electrophysiological data were not easily comparable due to the different recording time points: ERPs were recorded concurrently with the syllable of interest while behavioral data were collected substantially later. As a consequence, the classification of correct and incorrect trials is based on a more holistic estimation of the previously heard sequence (“does the whole sequence feel correct or incorrect?”) rather than on the evaluation of each particular syllable (“was the 11th syllable correct or incorrect?”) while the ERP response was specific to the individual syllables.

Given the late time window chosen for P3b analysis in the current experiment, one might argue that we were looking at a P600 rather than a P3b component. However, we argue that this would not affect our main conclusions for two reasons. First, we refrain from a strict distinction between P300 and P600. In line with others [24], [25], we assume that both components form part of the same ‘family’. The longer latency should result from the higher complexity of the stimulus characteristics, i.e. both physical properties (tones versus words) as well as structural properties (nested sentences versus linearly arranged words). Hence, we prefer to label the evoked component based on the complexity of the stimulus design. Comparing the current study design with those eliciting a P600 component (e.g. [26], [27]) the latter required implicit knowledge of formal stimulus characteristics while for the former it was sufficient to match a given sequence with a previously provided pattern. We are confident that the complexity of the current design would evoke a P3b rather than a P600. Second, we have shown previously that the P600, like the P3b, is sensitive to isochronous stimulus presentation [28] and depends on attention allocation [29]. Hence, even if the positivity evoked by the current design was a P600, this would not change our hypotheses or conclusions. A further aim of this study was to compare different methods of aligning language stimuli. As the p-center is considered to be more salient than physical stimulus onset, we hypothesized more efficient synchronization to the former than to the latter. In line with our expectations, we found a shorter P3b latency for the iso-PC condition than for both iso-SOA and jit-SOA conditions indicating more efficient deviant processing in p-center aligned syllable streams. This finding provides (as far as we know) first electrophysiological evidence for the relevance of the p-center concept in linguistic stimulus processing and hence opens up new perspectives on the establishment of temporal regularity in speech. This issue may be particularly important for future studies on disorders with speech processing deficits thought to arise from temporal processing deficits such as Parkinson's disease, basal ganglia lesions [30], [31], or stuttering [32]. Future work should assess whether patients with these perceptual disorders benefit from p-center-isochronous speech streams to overcome their specific linguistic processing deficit, and hence whether this concept has therapeutic implications. Furthermore, the current results may be relevant in the context of word learning: if p-center-aligned word lists attract attention in the same way as p-center-aligned syllables, they should result in better encoding and faster learning of new words compared with SOA-aligned word lists.

### Conclusion

In conclusion, we provide further evidence for the notion that auditory processing efficiency is a function of temporal predictability not only in simple tone sequences but also in linguistic stimulus streams. Varying temporal regularity of a stimulus stream modulated amplitude and latency of the P3b component that is associated with stimulus-related attention. In the isochronous conditions, attention was shifted to prospective occurrences of the relevant language stimuli entailing enhanced performance. The present results indicate not only that regular stimulus timing positively affects syllable processing as such but that the alignment of p-centers of linguistic stimuli results in faster stimulus evaluation.

## Supporting Information

Supporting Information S1 **Condition iso-PC incorrect.**
(WAV)

Supporting Information S2 **Condition iso-PC correct.**
(WAV)

Supporting Information S3 **Condition iso-SOA incorrect.**
(WAV)

Supporting Information S4 **Condition iso-SOA correct.**
(WAV)

Supporting Information S5 **Condition jit-SOA incorrect.**
(WAV)

Supporting Information S6 **Condition jit-SOA correct.**
(WAV)
